# Fabrication of anatomical sonographic phantoms through direct 3D-printing

**DOI:** 10.1093/bjro/tzag017

**Published:** 2026-08-12

**Authors:** Joanne Alice Zejmo, Laura Gorman, Christian Myles, James F X Jones

**Affiliations:** School of Medicine, University College Dublin, UCD Health Sciences Centre, Belfield, Dublin 4, D04 V1W8, Ireland; School of Medicine, University College Dublin, UCD Health Sciences Centre, Belfield, Dublin 4, D04 V1W8, Ireland; School of Medicine, University College Dublin, UCD Health Sciences Centre, Belfield, Dublin 4, D04 V1W8, Ireland; School of Medicine, University College Dublin, UCD Health Sciences Centre, Belfield, Dublin 4, D04 V1W8, Ireland

**Keywords:** 3D printing, ultrasound, phantom, interventional radiology, IR training

## Abstract

**Objectives:**

Sonographic anatomy classes can be curtailed by the availability of subjects for sensitive regional scanning. These limitations can be overcome by using anatomically realistic ultrasound phantoms (USPs). Simulation-based training with USPs also allows interventional radiology (IR) trainees to safely practice complex procedures. However, commercial phantoms are not patient-specific and involve costly, lengthy production methods. We describe a simple method for producing anatomically realistic USPs from 2D grey-scale ultrasound images using rapid direct 3D-printing technology.

**Methods:**

Original sonographic images were obtained from an open-source database and by scanning student volunteers. Images were digitally modified using Inkscape, ImageJ, Blender, Meshmixer, and CHITUBOX software programs. Models were printed using resin photopolymer 3D printers. In total, 4 phantoms were made: a femoral trochlea, a pediatric appendix, an eye with retinal detachment, and a radial nerve/brachioradialis model. The ImageJ Fiji plugin function, “Block Matching Correspondences” (BMCs) was used to assess similarity between source and phantom images.

**Results:**

All phantom images displayed BMCs with the original images (14%-43% similarity). The radial nerve model displayed the highest similarity. USPs produced with a hydrophilic swellable resin could be imaged throughout their full thickness and penetrated by a needle.

**Conclusions:**

In summary, this approach can manufacture USPs from 2D ultrasound data capable of generating anatomically accurate, patient-specific sonographic images. The low cost of these models, alongside their reproducibility through rapid 3D-printing technology, makes them a highly accessible, effective tool for simulation-based IR training.

**Advances in knowledge:**

To our knowledge, this study presents the first anatomical USPs, which have been directly 3D-printed from a single 2D ultrasound image source.

## Introduction

Interventional radiology (IR) procedures are often highly complex and demand accurate anatomical localization. As a result, there is a growing need for dependable training models.^1^ In order for IR trainees to safely perform ultrasound (US)-guided interventions, they require practice to develop relevant psychomotor abilities and hand-eye coordination.^2^ These skills can be achieved and maintained via simulation-based training.^2^ This form of training can involve phantoms and is playing an increasingly pivotal role in IR education, and improves upon the apprenticeship model of teaching by providing a standardized training approach with reduced variability.^2^

A phantom is a synthetic model of patient anatomy,^3^ composed of any material other than living human tissue.^1^ Training with ultrasound phantoms (USPs) improves patient safety by allowing trainees to practice US-guided procedures in a controlled, safe, and reproducible environment. This allows them to develop confidence and familiarity with interventions before attempting invasive procedures on live patients.^1^^,^^2^^,^^4^ Therefore, phantom-based training contributes to overall reduced anxiety, improved proficiency among novices, and lower risk of potentially life-threatening surgical complications.^2^ In addition, training with USPs does not require long-hours of expert vigilance and feedback when compared to training via supervised procedures.^1^ Traditional medical phantoms have been fabricated via manufacturing processes demanding expensive, time-consuming tool preparation, such as molding and casting. This high tooling cost has made it economically impractical to fabricate individualized phantoms for each patient.^5^ However, manufacturing technologies capable of producing 3-dimensional (3D) objects have accelerated in recent years, enabling the rapid prototyping of reproducible and sophisticated phantoms.^6^

3D printing, or additive manufacturing, involves a wide range of processes which combine diverse materials to create 3D objects from digital files.^7^^,^^8^ This technology can overcome the limitations of traditional manufacturing processes, and facilitate rapid production of low-cost, high-fidelity, patient-specific phantoms.^5^ The financial benefit of 3D-printed phantoms is particularly relevant for those intended to be destroyed or damaged during use, such as for practicing IR procedures.^3^ While 3D-printed phantoms have been widely produced and utilized in IR education, their creation from 2-dimensional (2D) US images remains under-explored. To date, the majority of physical models of patient anatomy derive from computed tomography (CT) data which is laboriously segmented until the desired structures are isolated using shared grey values (Hounsfield units). However, there is currently increasing interest in creating phantoms from other datasets such as ultrasound.^9^ Patient-specific phantom models recreated from US images have many potential roles in personalized medicine and can serve as valuable tools in medical education, by advancing practical training and theoretical teaching.^10^ Derivation of models from 3D ultrasound also usually involves a segmentation approach and digital model building.

The aim of this study is to design and produce accurate anatomical US phantoms from 2D grayscale US images, using direct 3D-printing and without segmentation or digital model building.

In order to effectively train healthcare providers to perform US-guided injections, a phantom must have similar echogenicity to human tissue when viewed on US.^4^ Furthermore, an ideal phantom should be inexpensive, capable of repeated use, readily available, provide tactile feedback, not be a health hazard, and be able to hold a needle in place.^1^^,^^4^^,^^11^

We hypothesized that it is possible to use 3D-printing after converting a single 2D US image into an accurate 3D USPs with grey-scale details closely resembling human tissue.

Importantly, the use of low-cost 3D-printing technology will ensure that phantoms will be time- and cost-effective to produce. The application of new photopolymers can permit the final product to be flexible, safe to use and capable of operating as a high-fidelity, anatomically accurate, and effective IR training phantom.

## Methods

**Figure 1:**
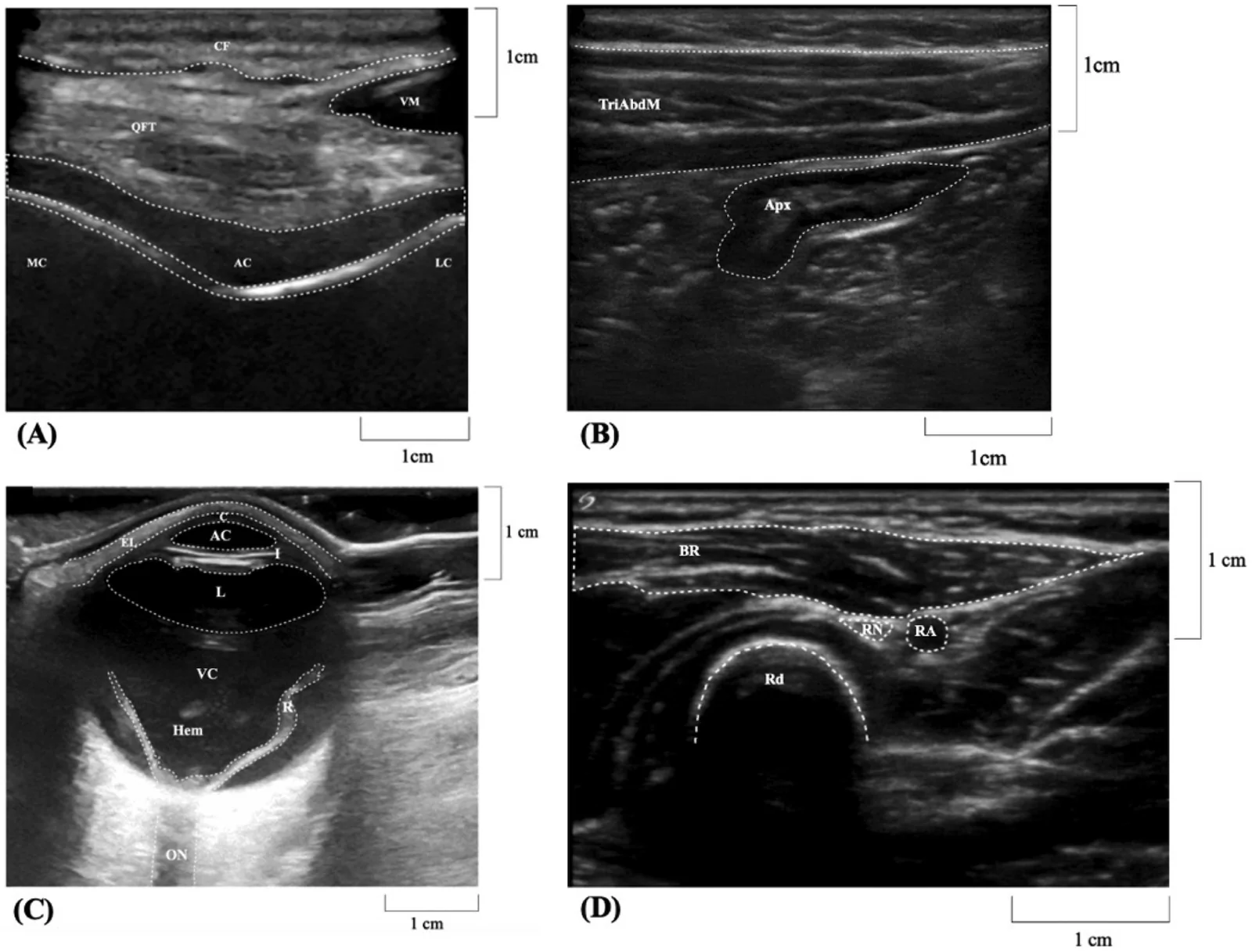
Original ultrasound images demonstrating relevant sono-anatomy. (A) Transverse view of the articular surface of the femoral trochlea. MC = Medial Condyle; AC = Articular Cartilage; LC = Lateral Condyle; QFT = Tendon of Quadriceps Femoris; VM = Vastus medialis muscle; CF = Crural fascia. Image taken by Joanne Zejmo, UCD School of Medicine. (B) Longitudinal view of normal pediatric appendix. Reference: “Normal Appendix- Blind End,” Dr. Sathya Subramaniam-Children’s Hospital of Philadelphia: THE POCUS ATLAS. (C) Longitudinal view of the eye with detached retina and vitreous hemorrhage. Reference: “Macula Off Retinal Detachment with Vitreous Hemorrhage,” Dr. Michael Kidon, PGY4, Denver Health Residency in Emergency Medicine: THE POCUS ATLAS. (D) Transverse view of radial nerve. Reference: “Radial Nerve,” Hannah Kopinski, Dr. Lindsay Davis-NYU Emergency Hospital; THE POCUS ATLAS. Abbreviations: AC = anterior chamber; AC = articular cartilage; Apx = appendix; BR = brachioradialis muscle; C = cornea; CF = crural fascia; EL = eyelid; Hem = hemorrhage; I = iris; L = lens; LC = lateral condyle; MC = medial condyle; ON = optic nerve; QFT = tendon of quadriceps femoris; R = retina; RA = radial artery; Rd = radius; RN = radial nerve; TriAbdM = trilaminar abdominal muscles; VC = vitreous chamber; VM = vastus medialis muscle

Original US images of the knee were obtained by scanning 4 student volunteers (*n* = 1 male, *n* = 3 female), using a Butterfly iQ + probe connected by USB-C cable to an Apple iPad, following institutional ethical approval (Figure 1A).

Each volunteer received an information leaflet, and consent form. None reported previous knee-related pathologies or surgery. Images were taken on Nerve preset, anonymized, and stored as PNG files on a Cloud-based system associated with the Butterfly iQ + system.

Other images were obtained from the free, open-access Creative Commons Attribution-NonCommercial 4. 0 International (CC BY-NC 4.0) imaging atlas “The POCUS ATLAS” (Figure 1B-D).

**Figure 2:**
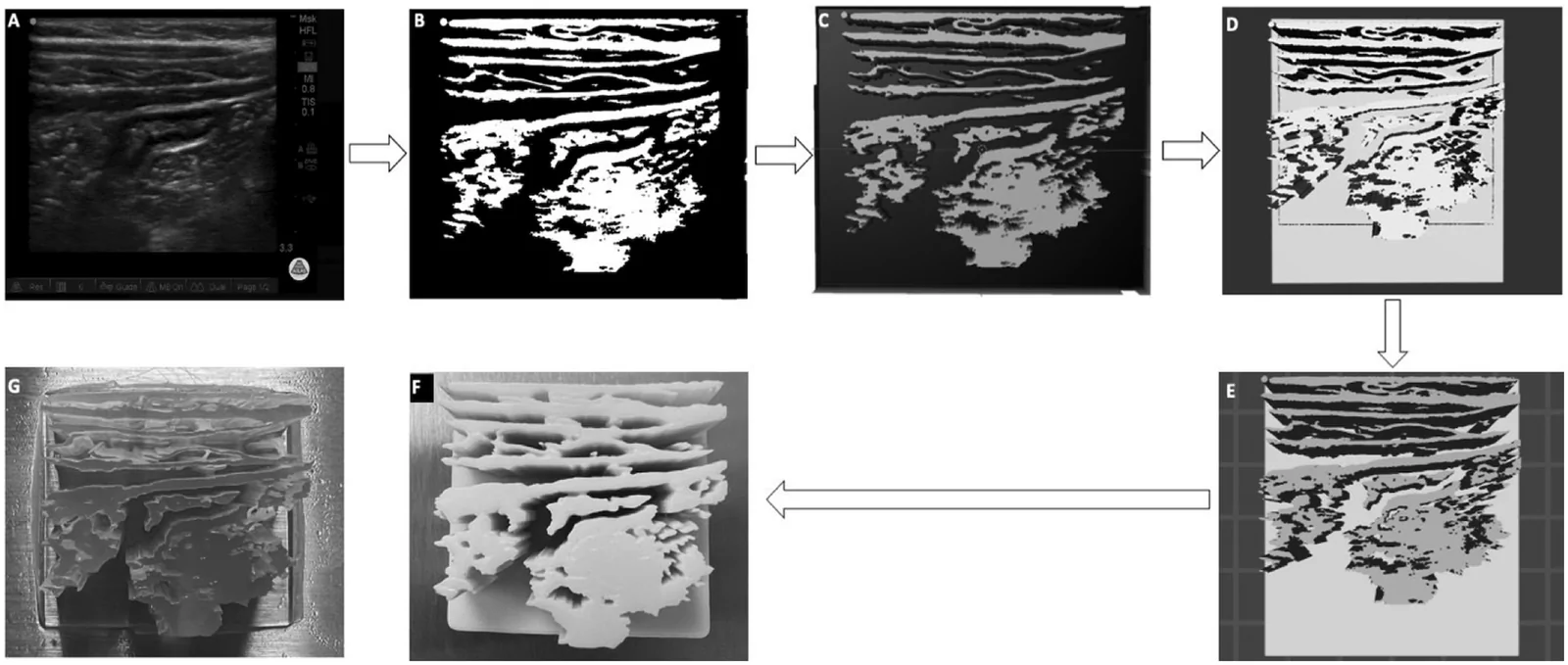
Schema for direct 3D-printing. (A) Original sonographic image of pediatric appendix was taken from open-access image atlas, The POCUS ATLAS, and saved as a PNG file. (B) PNG ultrasound image was modified using image processing programs Inkscape (Version 0.91 r13725) and ImageJ (Version 1.54 g). Modifications involved altering image contrast, and outlining the structure of anatomical features in the original image. (C) Modified images were extruded using Blender (Version 2.83) 3D graphics software. (D) Extruded models were edited and given a base using Meshmixer (Version 3.5.474) 3D modeling software, and saved as STL files. (E) Edited STL files were exported to CHITUBOX (Version 1.9.4) to be sliced and converted into g-code for printing. (F) Models were printed in Phrozen Aqua Ivory 4K Resin and in (G) Form Futura Engineering LCD Resin Flex, using a Masked Stereolithography (MSLA) resin 3D printer.

PNG US images were modified using the image processing programs Inkscape and ImageJ, modeled using Blender 3D graphics software, edited in Meshmixer, and exported into slicing software, CHITUBOX (Figure 2).

Phantoms were manufactured with Phrozen Aqua Ivory 4K Resin (Phrozen Technology, Shore Hardness = 80D, density (ρ) = 1.1 g/cm^3^), Form Futura Engineering LCD Series Flex 63A Resin (Form Futura, Shore Hardness = 82A, ρ = 1.18 g/cm^3^), and 3Dresyn SAE1 Swellable Absorbent and Elastic Hydrogel Resin (Table 1). Two Masked Stereolithography (MSLA) resin 3D printers were used for 3D printing, including the ELEGOO Saturn 2 (7980 × 4320 dpi, ELEGOO Saturn 2, Shenzhen, PRC) and ELEGOO Mars 4 Ultra (8520 × 4320 dpi, ELEGOO Mars 4, Shenzhen, PR China).

**Table 1:** Phantom production costs.

Phantom	Machine	Resin	Volume (mL)	Price (€)	Time
Femoral trochlea phantom	ELEGOO Saturn S	Form Futura Engineering LCD Series Flex Resin 82A:63A 1:2 v/v	9.09	0.73	2 h 50 min 35 s
Pediatric appendix phantom	ELEGOO Saturn S	Form Futura Engineering LCD Series Flex Resin 82A:63A 1:2 v/v	17.84	1.43	2 h 57 min 18 s
	ELEGOO Mars 4 Ultra	3Dresyn SAE1 Swellable Absorbent and Elastic Hydrogel Resin	17.84	13.16	2 h 28 min 43 s
Eye with retinal detachment and vitreous hemorrhage phantom	ELEGOO Saturn S	Form Futura Engineering LCD Series Flex Resin 82A:63A 1:2 v/v	40.14	1.11	2 h 55 min 32 s
Radial nerve phantom	ELEGOO Saturn S	Form Futura Engineering LCD Series Flex Resin 82A:63A 1:2 v/v	9.39	0.29	2 h 23 min 54 s
	ELEGOO Mars 4 Ultra	3Dresyn SAE1 Swellable Absorbent and Elastic Hydrogel Resin	9.39	6.93	2 h 4 min 30 s

### Printer settings

See Tables S1–S3.

### Post-processing

Printed models were submerged in an ELEGOO Mercury XS Wash Tank containing Isopropyl alcohol, cured in an ELEGOO Mercury XS Cure Machine, before being submerged in a degassed water container, scanned, and imaged.

**Figure 3:**
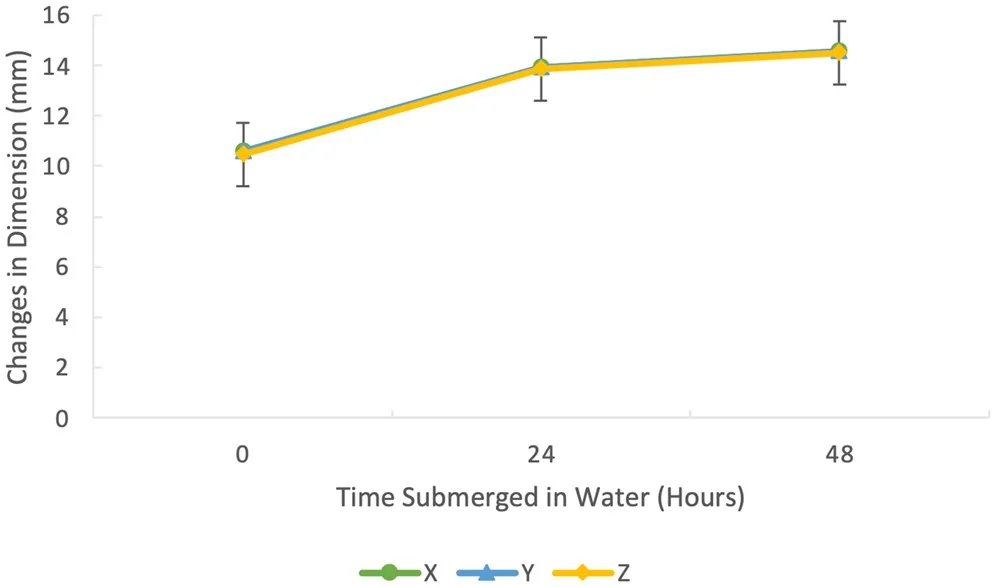
Degree of swelling of the 3Dresyn SAE1 swellable absorbent and elastic hydrogel resin. Five identical sample cubes (volume = 1 cm^3^) were printed on an ELEGOO Saturn 2 3D printer using 3Dresyn SAE1 Swellable Absorbent and Elastic Hydrogel Resin. The initial X, Y, and Z dimensions of each sample cube were recorded in millimeters prior to submersion in water, and subsequently re-measured at 24 and 48 h post-submersion. Changes in the X, Y, and Z dimensions of the sample cubes over time were recorded as average change in millimeters ± SD.

To quantify the degree of swelling of the 3Dresyn SAE1 Swellable Absorbent and Elastic Hydrogel Resin, 5 identical 3D-printed sample cubes with a volume of 1 cm^3^ were soaked in water for 48 h and measured periodically for changes in X, Y, and Z dimensions, as well as weight (Figure 3). Values were expressed as mean change in millimeters ± SD and mean change in grams ± SD respectively.

### Validation

Prior to image analysis an alignment of the 2 images was conducted (referred to as image registration by the software Fiji or Image J). This algorithm creates a spatial correspondence between subparts of the images and uses Pearson’s correlation to compute transformations for the whole image. This is normally used for alignment of serially sectioned histological tissues. Block matching calculates mean absolute differences in blocks of pixels and is normally used to highlight motion in sequential high frame rate videos. This cross fertilization of methods from different fields has allowed us to compare the source and derived images to test the validity of the simulation. We also considered a scale-invariant feature transform (SIFT) algorithm but it analyses grayscale feature information. As our method operates on simple binary (black and white) thresholding this was not suitable.

Prior to analysis, each phantom image was aligned to the original US image with which it was being compared to using the Fiji (version 1.54f) registration plugin “Align Image by line ROI.” First, the original US image, which served as the template, was loaded into ImageJ and 2 landmarks were specified by line selection. Next, the phantom image was imported and a line selected such that its first point correlated with the first point of the template image’s line selection. After the plugin had been run, the feature extraction plugin “Test Block Matching Correspondences” was applied. This feature initially performs an approximate linear alignment between images based on local features, followed by local vicinity inspection around each feature point, or vertex, to find optimal matches between the 2 images. Two other image analysis methods were compared to this approach. Values obtained from this comparison of image analysis methods were expressed as mean ± SD (Table 2).

**Table 2:** Potential model validation methods control trial—SSIM, average pixel difference, block matching correspondences.

Comparison	Structural similarity index measure	Average pixel percentage difference	Block matching correspondences—match points/block match candidates
A–B	0.15	14	0/221
A–C	0.25	13	0/237
A–D	0.14	13	0/236
B–C	0.17	27	0/175
B–D	0.09	76	0/198
C–D	0.11	35	0/205
Mean (SD)	0.15 (0.06)	30 (24)	0 (0)

**Figure 4:**
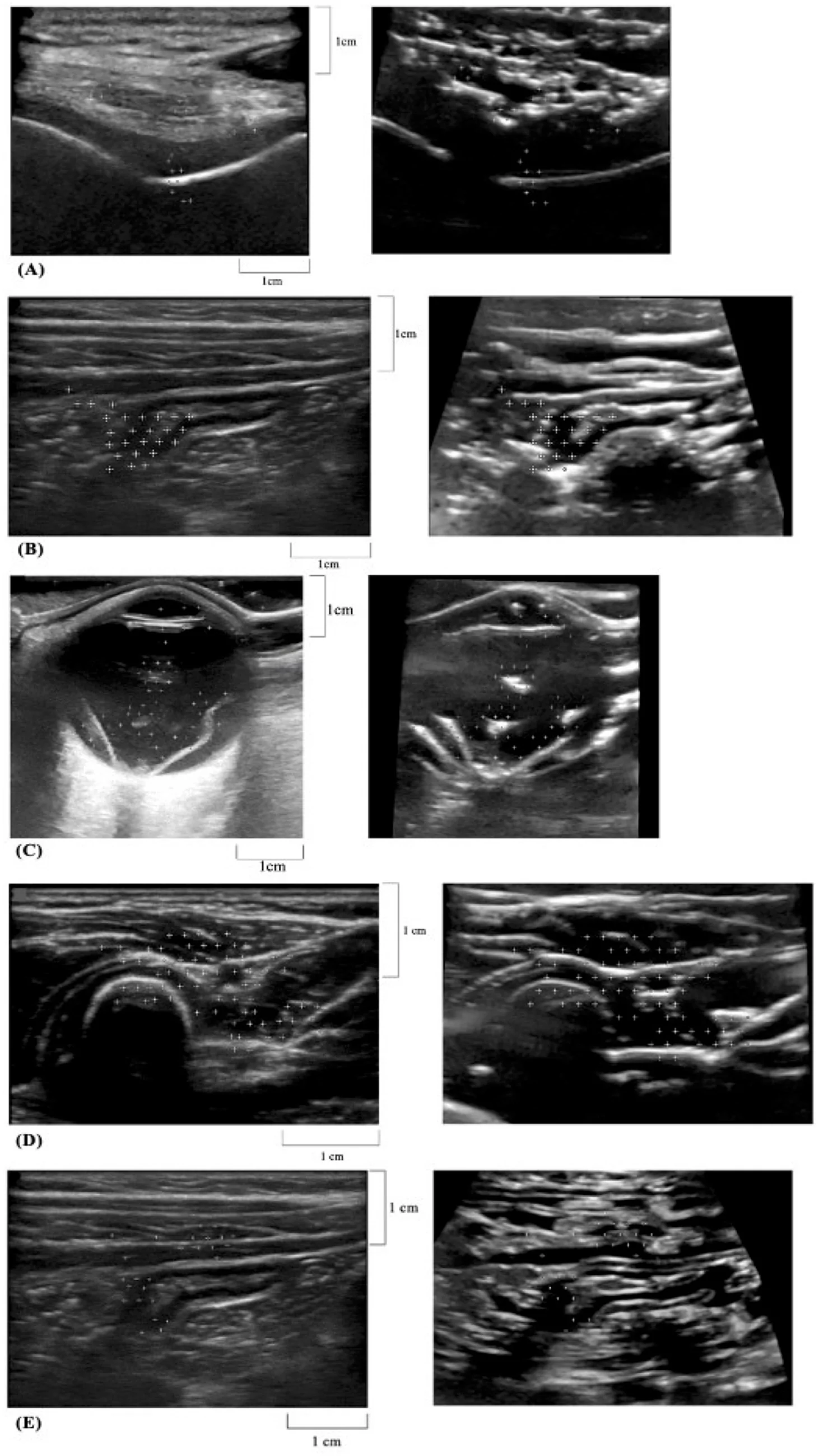
Results of “Block Matching Correspondences.” White crosses: Match points. Original ultrasound images were imported to Fiji (Version 1.54f), scaled, and aligned to a region of interest, before applying the feature extraction plugin “Block Matching Correspondences” to assess similarity. (A) Original image of femoral trochlea (left) and phantom (right) were found to have 26 match points out of 191 block match candidates. (B) Original image of pediatric appendix (left) and respective phantom displayed 26 match points out of 108 block match candidates. (C) Image of eye with retinal detachment and vitreous hemorrhage (left) had 66 match points with ocular phantom (right) out of 260 block match candidates. (D) Original image of radial nerve (left) and respective phantom (right) displayed 72 match points out of 169 block match candidates. (E) Original image of pediatric appendix (left) and phantom (right) printed with 3Dresyn SAE1 Swellable Absorbent and Elastic Hydrogel Resin displayed 29 match points out of 237 block match candidates.

Original images (displayed on the left side of Figure 4A–D, with newly acquired model images adjacent), were converted to 300 dpi TIF files before being imported to Fiji (Version 1.54f) and compared using SSIM, Average Pixel Difference, and Block Matching Correspondences. A—Appendix, B—Eye, C—Femoral Trochlea, D—Radial Nerve.

## Results

First, a test of similarity indices was performed using the original images from “THE POCUS ATLAS” (CC BY-NC 4.0), to compare the validity of 3 image comparison algorithms within Fiji (Version 1.54f), to determine which would be most appropriate for comparing template images with images of their respective phantom. The 3 algorithms evaluated included the plugins “Structural Similarity Index Measure” (SSIM), and “Block Matching Correspondences” (BMCs), in addition to the image calculator option ‘Subtract’ which provided average pixel percentage difference between 2 images. In the control trial, these algorithms were each used to compare 6 possible combinations of the 4 original US images upon which the phantoms were based. Given that the original US images are of different anatomical structures, they should not display any matching when compared. Only the “Block Matching Correspondences” plugin resulted in an average match of 0% ±0 SD between unrelated original US images, thus we selected it to perform our image comparisons for the remainder of the study.

Phantoms printed in Form Futura Engineering LCD Series Flex Resin 82A:63A mix (1:2 v/v) were assessed for similarity with the original image from which they were constructed (Figure 4). After extracting block matching correspondences, the original image of the femoral trochlea and its phantom were shown to have 26 match points out of 191 block match candidates, resulting in a 13.6% match. The original image of the pediatric appendix and its respective phantom were found to have 26 match points out of 108 block match candidates. This resulted in a 24.1% match, indicating a greater level of similarity between original image and phantom when compared to the femoral trochlea model and its source image. Next, the original US image of the eye with retinal detachment and vitreous hemorrhage was compared to an image of its phantom, resulting in 66 match points out of 260 block match candidates. This revealed the second highest level of similarity between source image and phantom, with a 25.4% match.

Lastly, the original image of the radial nerve was compared to its phantom printed in Form Futura Engineering LCD Series Flex Resin 82A:63A mix (1:2 v/v), producing 72 match points out of 169 block match candidates. This 42.6% match indicated that the radial nerve phantom printed with Form Futura Engineering LCD Series Flex Resin 82A:63A mix (1:2 v/v) had the highest level of similarity when compared to the US image used as its template.

When the original image of the pediatric appendix was compared to its phantom printed with 3Dresyn SAE1 Swellable Absorbent and Elastic Hydrogel Resin, a total of 29 match points was found for 237 block match candidates. This 12.2% match was lower than the 24.1% match found when the original image of the pediatric appendix was compared to its phantoms printed in Form Futura Engineering LCD Series Flex Resin 82A:63A mix (1:2 v/v).

After the 5 sample cubes printed to quantify the degree of swelling of the 3Dresyn SAE1 Swellable Absorbent and Elastic Hydrogel Resin were submerged in water for 24 h, their average weight increased by 89%-95% from 1.48 to 2.84 g. After being submerged for 48 h, the average weight of the 5 cubes increased by an overall 120%-124% when compared to their original average weight, to 3.29 g. Of note, the 5 sample cubes demonstrated isotropic swelling in all directions.

## Discussion

To our knowledge, this study presents the first anatomical USP which have been directly 3D printed from a single 2D US image. The phantom images were compared to the original source image using 3 different algorithms. It was found that of the 3 popular similarity indices only one reported zero similarity when unrelated images were compared. Therefore, the most robust method (block matching comparisons) was applied to the analysis of all phantoms. This approach not only quantifies overall image similarity but also highlights where in the phantom image the best correspondence to the source image is achieved. This is a valuable tool for designers of simulated tissue, particularly when there is a target (eg, nerve) which must be reached by an instrument.

This is the first study to compare a phantom to its sonographic source (as it is the basis of the novel method). To the authors’ knowledge, our conservative block matching similarity index is the only similarity measure that has been applied to any sonographic phantom. Commercial phantoms cannot be analyzed in this manner as they are not derived from, or spatially aligned to, unique real images. A block matching analysis applied injudiciously would yield a similarity of zero for other phantoms.

One limitation of the phantoms produced with Form Futura Engineering LCD Series Flex Resin was their hardness, which limited the transmission of sound throughout the entire thickness of the model. We believe images obtained from scanning the phantoms printed with this resin were produced via the reflection of US waves from the superficial edge facets of each model. As a result, while phantoms made with Form Futura Engineering LCD Series Flex Resin generated detailed images, they were not suitable as IR training models, as their interior structure could not be visualized by users when attempting needle placement.

This was overcome by printing the models with 3Dresyn SAE1 Swellable Hydrogel Resin, which facilitated the transmission of sound through their full thickness after being stored in water for around 72 h. However, this resin was prone to some delamination when stored. This may limit the durability and reusability of the phantoms for practicing IR procedures which would induce mechanical stress from repeated needle punctures and multiple submersions in water.

**Figure 5:**
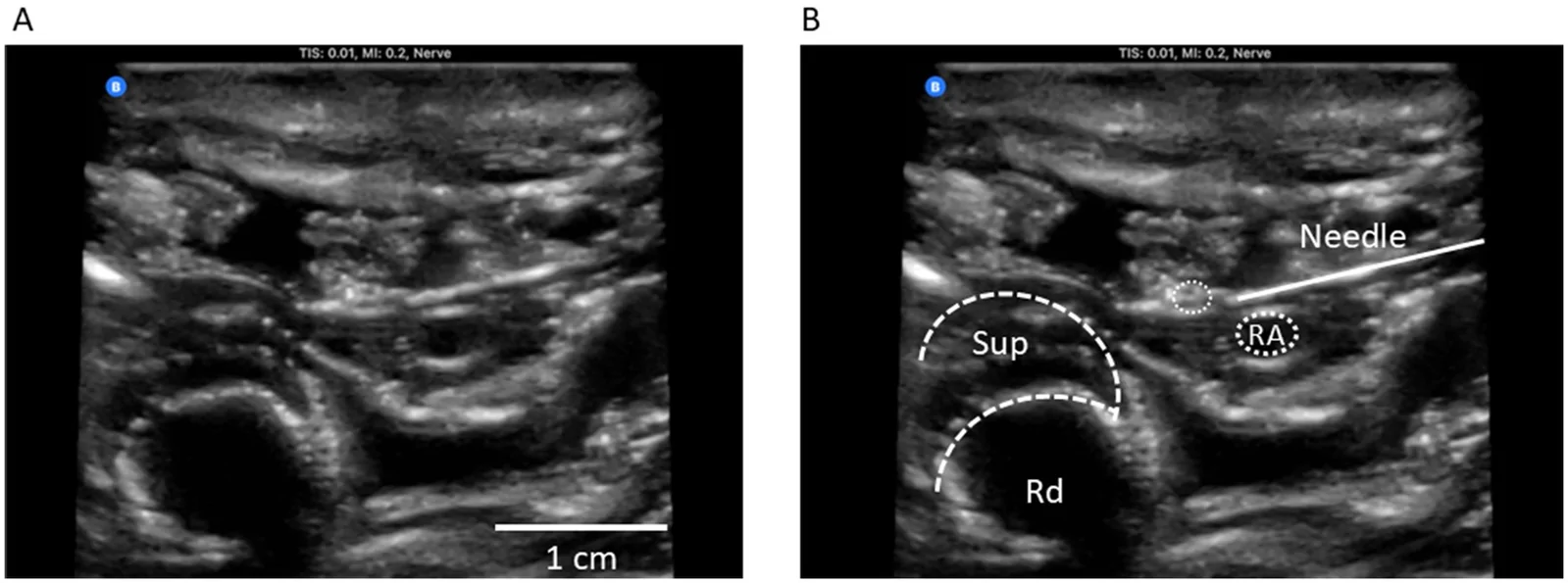
A hydrogel resin print of radial nerve model in forearm showing needle insertion. The radial nerve model was digitally diminished to 70% before printing and after 24 h post print hydration it regained its original proportions. A needle was inserted to target the radial nerve. Panel B is the labeled version of panel A. Abbreviations: RA = radial artery; Rd = neck of radius; Sup = supinator.

In addition, this elastic hydrogel resin displayed significant swelling, which resulted in model expansion. This may have contributed to the reduction in similarity observed between the original image of the pediatric appendix and its phantom printed in this material, versus the Form Futura Engineering Flex Resin. This limitation can be overcome in future experiments with the development of more specialized photopolymer resins. However, the swelling of the current hydrogel was isotropic in X, Y, and Z axes and therefore can be overcome by simply contracting the printable object by the appropriate fraction (approx. 70%, see Figures 3 and 5) before manufacturing.

Potential future applications of our models include use in ultrasound “Spotter-tests,” where trainees can enhance their ultrasound and anatomy knowledge by scanning various model types to identify an array of anatomical structures and pathological conditions. An apparatus to secure individual phantoms while scanning in a water bath has been developed for this purpose (Figure S1).

Regarding future goals for use of the models in IR procedural training, a model where injection of simulated anesthetics with visual air and fluid retention is possible, as well as hydro dissection of fascial planes and perineural injection of fluid. These models may prove useful to the field of simulation medicine.

## Future directions

**Figure 6:**
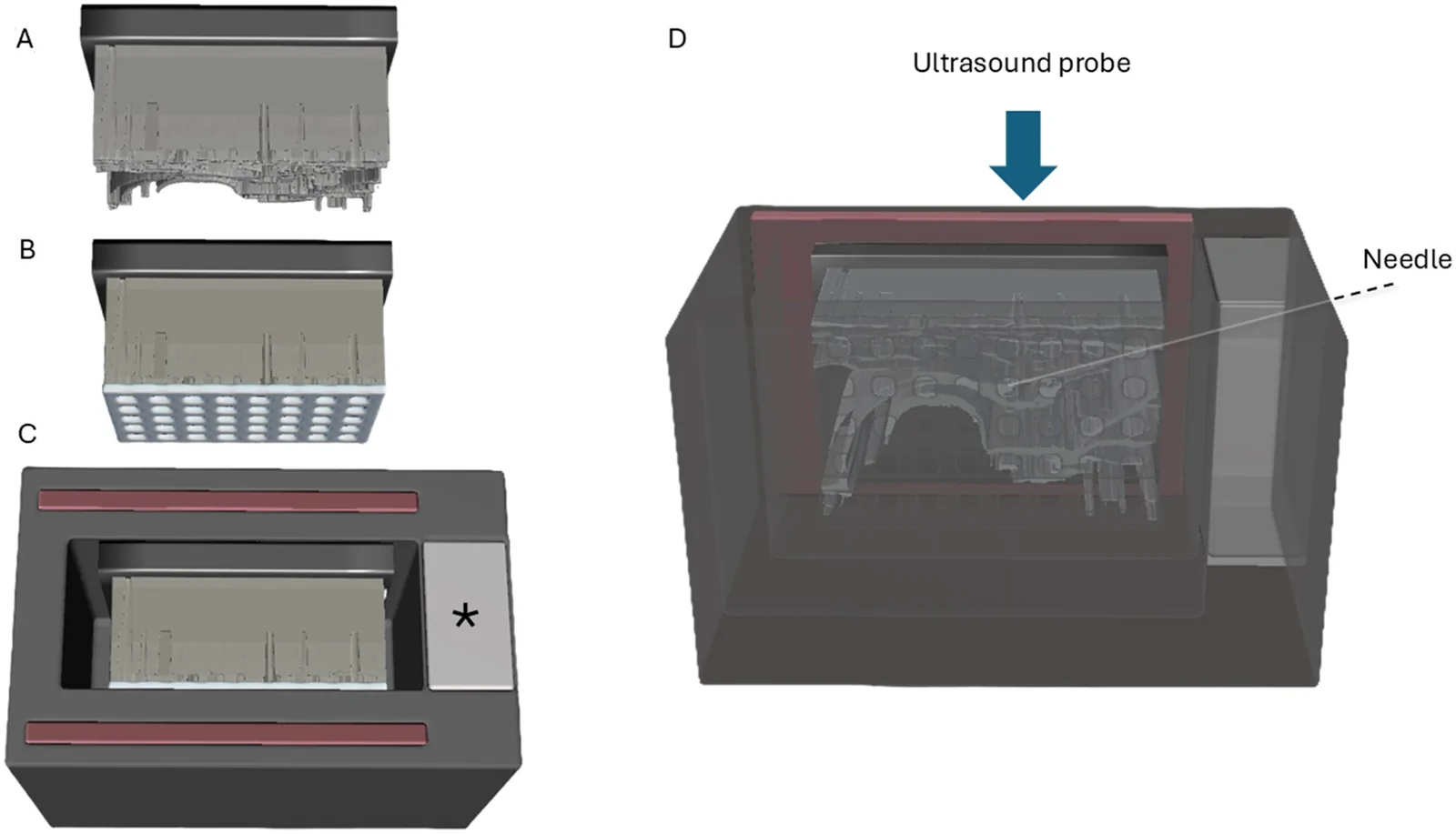
Digital design of a cassette holder for ultrasound phantoms. The extruded phantom (A) is modified to include a sieve (B) structure which permits resin to drain but also stabilizes the delicate hydrogel. This is inserted into a printed water bath (C) which contains a slot for a silicone slab * which acts as a needle port. Haptic feedback during needle insertion is optimized by adjusting thickness and shore hardness of the silicone (D).

A digital design to house the hydrogel phantoms is illustrated in Figure 6. It contains a needle port which will offer tunable haptic feedback for the operator. We have previously published on a silicone access system for vascular cannulation.^11^ Refinement of this system will require biomechanical tests and mass production in collaboration with radiologists who will be able to select the phantom image of interest. This is the topic of a future study.

## Conclusion

In conclusion, the approach described enabled us to produce an array of USPs from 2D ultrasound data via direct 3D printing, which were capable of generating accurate sonographic images and reproducing unique features of individual patient anatomy. The inexpensive nature of these models, combined with their reproducibility and potential to facilitate needle placement and biopsy procedure practice may make them a highly effective tool for simulation-based IR training and for practitioners using ultrasound-guided techniques for regional anesthesia.

## Supplementary Material

tzag017_Supplementary_Data

## References

[1] Qurash M , Yaacob N, Azuan N, Khaleel Y, Zakaria R. Special ultrasound phantom for interventional training: construction, advantages, and application. J Med Ultrasound. 2018;26:210-214. 10.4103/jmu.jmu_40_18 30662153 PMC6314090

[2] Giannotti E , Jethwa K, Closs S, et al Promoting simulation-based training in radiology: a homemade phantom for the practice of ultrasound-guided procedures. Br J Radiol. 2022;95:20220354. 10.1259/bjr.20220354 35856798 PMC10996965

[3] Silvestro E , Betts KN, Francavilla ML, Andronikou S, Sze RW. Imaging properties of additive manufactured (3D printed) materials for potential use for Phantom models. J Digit Imaging. 2020;33:456-464. 10.1007/s10278-019-00257-5 31520278 PMC7165207

[4] Kim YH. Ultrasound phantoms to protect patients from novices. Korean J Pain. 2016;29:73-77. 10.3344/kjp.2016.29.2.73 27103961 PMC4837122

[5] Wang K , Ho C-C, Zhang C, Wang B. A review on the 3D printing of functional structures for medical phantoms and regenerated tissue and organ applications. Engineering (Beijing). 2017;3:653-662. 10.1016/j.eng.2017.05.013

[6] Filippou V , Tsoumpas C. Recent advances on the development of phantoms using 3D printing for imaging with CT, MRI, PET, SPECT, and ultrasound. Med Phys. 2018;45:e740-e760. 10.1002/mp.13058 29933508 PMC6849595

[7] Li B , Zhang M, Lu Q, et al Application and development of modern 3D printing technology in the field of orthopedics. Biomed Res Int. 2022;2022:8759060. 10.1155/2022/8759060 35211626 PMC8863440

[8] Jandyal A , Chaturvedi I, Wazir I, Raina A. Ul Haq MI. 3D printing—a review of processes, materials and applications in industry 4.0. Sustain Oper Comput. 2022;3:33-42. 10.1016/j.susoc.2021.09.004

[9] Ripley B , Levin D, Kelil T, et al 3D printing from MRI data: harnessing strengths and minimizing weaknesses. J Magn Reson Imaging. 2017;45:635-645. 10.1002/jmri.25526 27875009

[10] Nilsson DP , Holmgren M, Holmlund P, et al Patient-specific brain arteries molded as a flexible phantom model using 3D printed water-soluble resin. Sci Rep. 2022;12:10172. 10.1038/s41598-022-14279-7 35715506 PMC9205921

[11] O’Reilly MK , Reese S, Herlihy T, et al Fabrication and assessment of 3D printed anatomical models of the lower limb for anatomical teaching and femoral vessel access training in medicine. Anat Sci Educ. 2016;9:71-79. 10.1002/ase.1538 26109268

