# Supplementary material for: Fabrication of anatomical sonographic phantoms through direct 3D-printing
Source: BJR Open. 2026 Aug 12;8(1):tzag017. doi: 10.1093/bjro/tzag017 (PMC13563663; doi:10.1093/bjro/tzag017)
Supplement: Supplementary file 1 [file tzag017_supplementary_data.zip › Supplementary Table S3.pdf]

Table S3: Printer Settings- 3Dresyn SAE1 Swellable Absorbent and Elastic Hydrogel Resin

Layer Height	0.050 mm	Bottom Lift Distance	3.000 mm
Bottom Layer Count	4	Lifting Distance	3.000 mm
Exposure Time	10.000 s	Bottom Retract Distance	3.000 mm
Bottom Exposure Time	100.000 s	Retract Distance	3.000 mm
Transition Layer Count	10	Bottom Lift Speed	60.000 mm/min
Transition Type	Linear	Lifting Speed	60.000 mm/min
Transition Layer Interval Time Difference	8.18 s	Bottom Retract Speed	60.000 mm/min
Waiting Mode During Printing	Light off delay	Retract Speed	60.000 mm/min
Light-off Delay	0.700 s	Matching Resin Mode	Standard Resin_Normal
Bottom Light-off Delay	0.000 s		
