# Supplementary material for: Fabrication of anatomical sonographic phantoms through direct 3D-printing
Source: BJR Open. 2026 Aug 12;8(1):tzag017. doi: 10.1093/bjro/tzag017 (PMC13563663; doi:10.1093/bjro/tzag017)
Supplement: Supplementary file 1 [file tzag017_supplementary_data.zip › Supplementary Figure S1.pdf]

II. Figure S1: Adjustable Phantom Holder with Clips

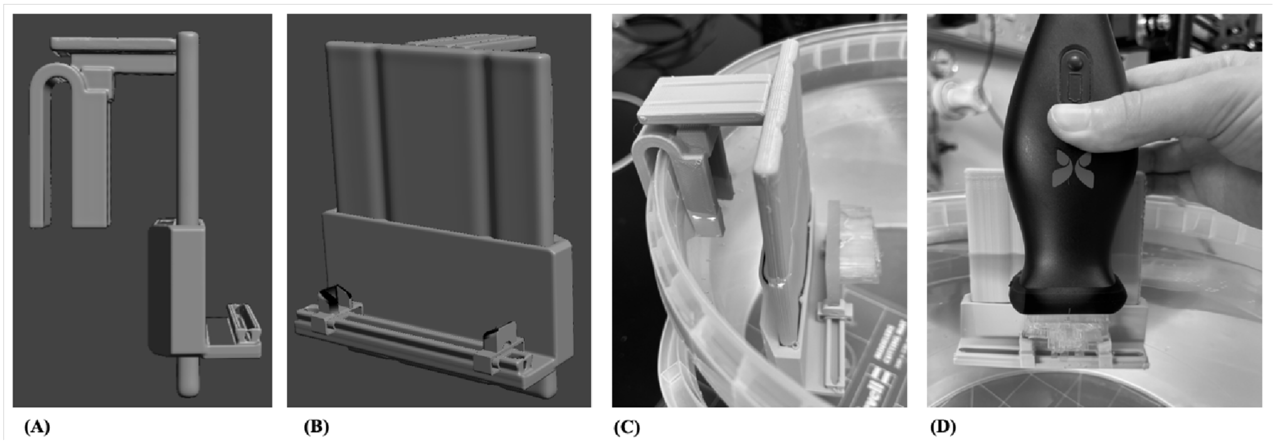

Figure S1: Adjustable Phantom Holder with Clips. Digital model of the phantom holder was designed in Meshmixer^{TM} (Version 3.5.474), shown in (A) side-view and (B) frontal view with moveable clips for securing phantom attached to track. (C) Holder attached to water bucket, with a phantom in place. (D) Demonstration of scanning a phantom using the Butterfly iQ^{TM} probe, with support of the holder.
