# Supplementary material for: Fabrication of anatomical sonographic phantoms through direct 3D-printing
Source: BJR Open. 2026 Aug 12;8(1):tzag017. doi: 10.1093/bjro/tzag017 (PMC13563663; doi:10.1093/bjro/tzag017)
Supplement: Supplementary file 1 [file tzag017_supplementary_data.zip › Supplementary Table S1.pdf]

<u>Supplementary: Fabricating anatomical sonographic phantoms through direct 3D printing</u>

Table of Contents for Supplementary:

I. Table S1-S3: Printer Settings
II. Figure S1: Adjustable Phantom Holder with Clips

I. Table S1-S3: Printer Settings

<u>Table S1: Printer Settings- Phrozen Aqua Ivory 4K Resin</u>

Layer Height	0.050 mm	Bottom Lift Distance	10.000 mm
Bottom Layer Count	8	Lifting Distance	10.000 mm
Exposure Time	3.000 s	Bottom Retract Distance	10.000 mm
Bottom Exposure Time	30.000 s	Retract Distance	10.000 mm
Transition Layer Count	6	Bottom Lift Speed	60.000 mm/min
Transition Type	Linear	Lifting Speed	60.000 mm/min
Transition Time Decrement	3.860 s	Bottom Retract Speed	240.000 mm/min
Light-off Delay	2.500 s	Retract Speed	150.000 mm/min
