# Supplementary material for: Fabrication of anatomical sonographic phantoms through direct 3D-printing
Source: BJR Open. 2026 Aug 12;8(1):tzag017. doi: 10.1093/bjro/tzag017 (PMC13563663; doi:10.1093/bjro/tzag017)
Supplement: Supplementary file 1 [file tzag017_supplementary_data.zip › Supplementary Table S2_.pdf]

Table S2: Printer Settings- Form Futura Engineering LCD Series Flex Resin (82A: 63A of 1:2 v/v mix)

Layer Height	0.050 mm	Bottom Light-off Delay	1.000 s
Bottom Layer Count	8	Bottom Lift Distance	8.000 mm
Exposure Time	8.000 s	Lifting Distance	6.000 mm
Bottom Exposure Time	40.000 s	Bottom Retract Distance	8.000 mm
Transition Layer Count	6	Retract Distance	6.000 mm
Transition Type	Linear	Bottom Lift Speed	40.000 mm/min
Transition Time Decrement	4.750 s	Lifting Speed	75.000 mm/min
Waiting Mode During Printing	Light-off Delay	Bottom Retract Speed	45.000 mm/min
Light-off Delay	1.000 s	Retract Speed	45.000 mm/min
